# Serotonin and YAP/VGLL4 Balance Correlated with Progression and Poor Prognosis of Hepatocellular Carcinoma

**DOI:** 10.1038/s41598-018-28075-9

**Published:** 2018-06-27

**Authors:** Bo Shu, Mimi Zhai, Xiongying Miao, Chao He, Chaolin Deng, Yu Fang, Ming Luo, Luyao Liu, Sushun Liu

**Affiliations:** 1 Department of General Surgery, the Second Xiangya Hospital, Central South University, Changsha, Hunan 410011 China; 2 Department of Hepatobiliary Surgery, the First Affiliated Hospital of Xi’an Jiaotong University, Xi’an, Shaanxi 710061 China

## Abstract

YAP-TEAD complex plays an important role in tumorigenesis. 5-HT is proved to upregulate YAP expression by our previous study and VGLL4 is found to compete with YAP for binding to TEAD in several of cancers. Here, we investigated whether 5-HT could affect progression and prognosis of hepatocellular carcinoma (HCC) patients and regulate YAP/VGLL4 balance. We found that 5-HT and YAP/VGLL4 ratio were higher in HCC patients and closely related with progression and poor prognosis. Furthermore, 5-HT level, YAP/VGLL4 ratio and tumor size were proved as independent risk factors of HCC patients in our study. Based on the independent risk factors, nomogram was established to exactly predict prognosis of HCC patients. Additionally, the study revealed that a higher total point of the nomogram was closely correlated with poorer prognosis. As a result, 5-HT might contribute to the progression and poor prognosis of hepatocellular carcinoma via regulating YAP/VGLL4 balance. Therefore, the established nomogram based on the independent risk factors may become an important part of HCC prediction system and YAP/VGLL4 balance may be a potential therapeutic target in future.

## Introduction

In China, hepatocellular carcinoma (HCC) reminds the sixth common malignancy and causes the third mortality among different kinds of cancer^1^. The morbidity of HCC is still increasing every year with poor prognosis. Serotonin (5-HT), a potent mitogen, is proved to be closely associated with liver regeneration as well as hepatocarcinogenesis^2,3^. Increasing evidence has indicated that serotonin promotes proliferation and invasion of hepatoma cell^3,4^. Besides, it has been proved to promote proliferation of xenograft in models^3,4^.

Landmark studies have implicated that YAP plays an essential role in promoting organ size and tumorigenesis^5–7^. Our previous study also demonstrate that serotonin promotes malignant biological behavior of hepatocellular carcinoma via regulation of YAP. In addition, a 5-HT-5-HT_2B_ receptor-pERK-YAP axis emerged out in this study^7^. Increasing number of studies indicate that YAP interactes with TEADs to activate target genes though it does not contain any DNA-binding domains. VGLL4, a novel tumor suppressor, is reported to compete with YAP for pairing with TEADs to disrupt the YAP-TEADs complex formation^8–11^. Further study conducted by Jiao *et al*. indicated that both YAP and VGLL4 levels should maintain a suitable balance or ratio to control organ development. Disrupting the homeostasis might lead to dysplasia or cancers^8^. Moreover, Jiao *et al*. demonstrated that YAP/VGLL4 ratios was sharply upregulated and closely correlated with tumor progress in gastric carcinoma. At present, YAP/VGLL4 balance has been studied in gastric carcinoma, esophageal squamous cell carcinoma, lung cancer and colorectal cancer, but not in HCC so far^8,9,11,12^. Based on our previous study, serotonin has the ability to upregulate the expression of YAP^7^. However, whether YAP/VGLL4 balance is regulated by serotonin remains unknown. Therefore, there is great need to identify the effects of YAP/VGLL4 balance on HCC and the regulation of it induced by serotonin.

In this work, we set out to investigate the whether serotonin is correlated with the prognosis of HCC via regulating YAP/VGLL4 balance, aiming to establish a nomogram prognosis prediction system of HCC as well as developing a potential therapeutic target for HCC patients.

## Results

### The 5-HT level was higher in HCC patients

**Figure 1:**
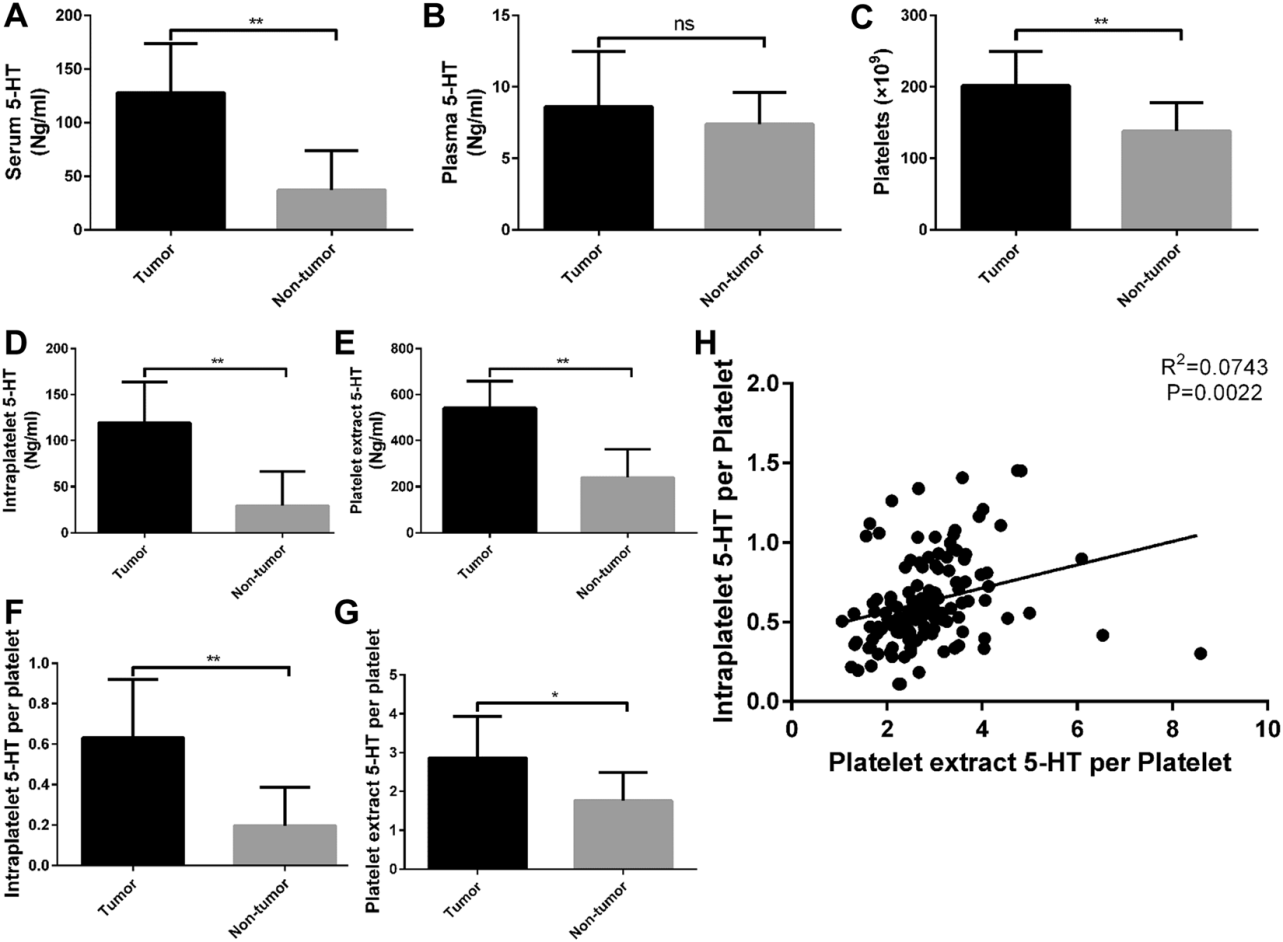
5-HT level was higher in HCC patients compared with non-tumor patients. (**A**,**B**) 5-HT level in serum was higher in HCC patients (**A**) but 5-HT level in plasma was not statistically significant (**B**). (**C**) The PLT levels was higher in HCC patients compared with non-tumor patients. (**D**–**G**) The intraplatelet 5-HT level (**D**) platelet extract 5-HT level (**E**) intraplatelet 5-HT per platelet (**F**) and platelet extract 5-HT per platelet (**G**) were all higher in HCC patients. (**H**) The platelet extract 5-HT per platelet and intraplatelet 5-HT per platelet were positively correlated. ns: no statistical significance, *P < 0.05, **P < 0.01.

First, we investigated the 5-HT level in serum and plasma, blood platelet count (PLT) in selected patients. Notably, the 5-HT levels in serum were higher in HCC patients as well as the PLT levels (Fig. 1A,C). The 5-HT level in plasma were also higher in HCC patients, but it was not statistically significant (Fig. 1B). In addition, the intraplatelet 5-HT level, platelet extract 5-HT level, intraplatelet 5-HT per platelet and platelet extract 5-HT per platelet were all higher in HCC patients than those in non-tumor patients (Fig. 1D–G). Moreover, the intraplatelet 5-HT per platelet and platelet extract 5-HT per platelet were positively correlated in HCC patients with no doubt (Fig. 1H).

### YAP expression upregulated in HCC patients and was positively correlated with 5-HT level

**Figure 2:**
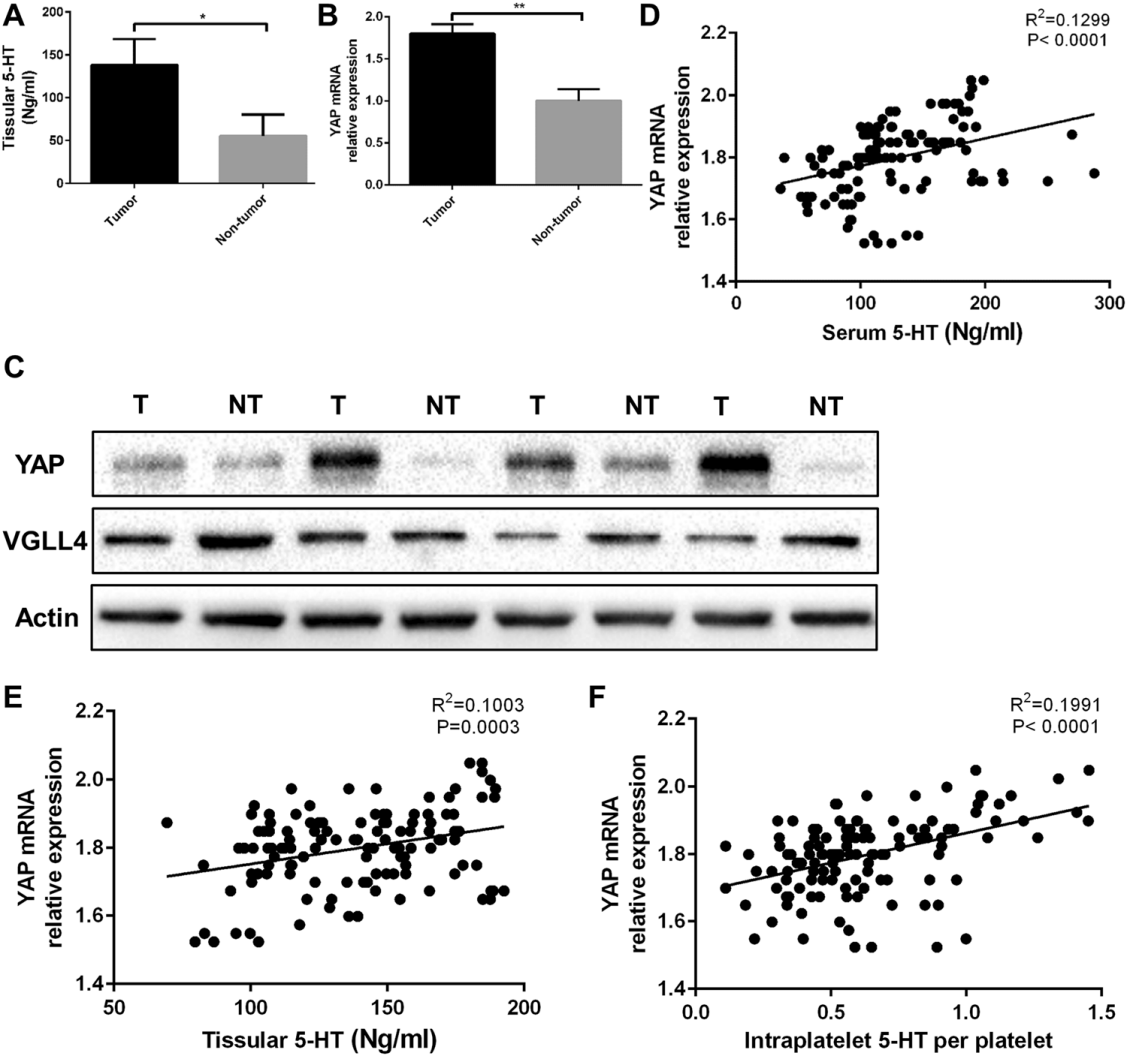
YAP expression upregulated in HCC patients and was positively correlated with 5-HT level. (**A**) 5-HT level in tissue samples of HCC patients were higher compared with non-tumor patients. (**B**,**C**) YAP expression of HCC patients in mRNA (**B**) and protein level (**C**) both upregulated. (**D**–**F**) The serum 5-HT (**D**) tissular 5-HT (**E**) and intraplatelet 5-HT per platelet (**F**) were both positively correlated with YAP expression in mRNA level. ns: no statistical significance, *P < 0.05, **P < 0.01.

We assessed the tissular 5-HT level and YAP expression in tissue samples as well. As expected, tissular 5-HT level was higher in HCC which was similar to serum 5-HT level (Fig. 2A). The YAP expression in mRNA and protein level were both upregulated in HCC patients (Fig. 2B,C). Furthermore, correlation analysis was conducted between 5-HT level and YAP expression. As a result, the serum 5-HT level, tissular 5-HT level and intraplatelet 5-HT per platelet were all positively correlated with YAP expression in tissue samples of HCC patients (Fig. 2D–F).

### VGLL4 expression downregulated in HCC patients and was negatively correlated with 5-HT level

**Figure 3:**
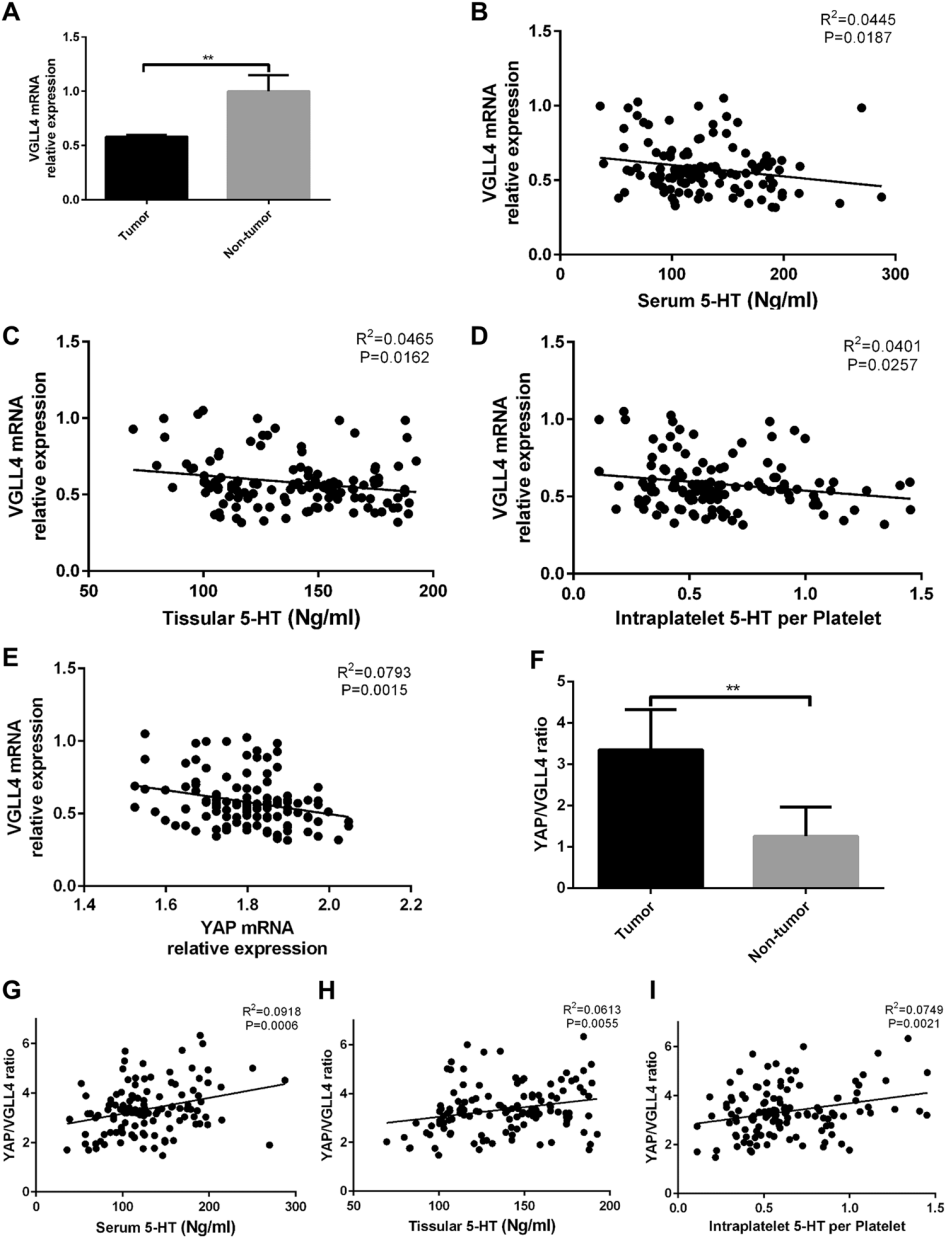
YAP/VGLL4 ratio upregulated in HCC patients and was positively correlated with 5-HT level. (**A**) VGLL4 expression in tissue samples of HCC patients were lower compared with non-tumor patients. (**B**–**D**) Serum 5-HT (**B**) tissular 5-HT (**C**) and intraplatelet 5-HT per platelet (**D**) were all negatively corrected with VGLL4 expression. (**E**) YAP expression and VGLL4 expression were negatively corrected. (**F**) YAP/VGLL4 ratio was higher in HCC patients. (**G**–**I**) Serum 5-HT (**G**) tissular 5-HT (**H**) and intraplatelet 5-HT per platelet (**I**) were all positively corrected with YAP/VGLL4 ratio. ns: no statistical significance, *P < 0.05, **P < 0.01.

To confirm the biological role of VGLL4 in HCC, we measured the VGLL4 expression in tissular samples of HCC patients. Both of the mRNA and protein level of VGLL4 downregulated in HCC patients (Figs 2C and 3A). To examine whether VGLL4 expression was correlated with 5-HT level, we performed several correlation analysis. As shown in Fig. 3B, the serum 5-HT level and VGLL4 mRNA relative expression were negatively correlated. Similar results were obtained in tissular 5-HT and intraplatelet 5-HT per platelet (Fig. 3C,D).

### YAP/VGLL4 ratio was correlated with HCC prognosis and 5-HT level

Because VGLL4 has the competitive inhibition effects on YAP-TEAD complex, we explored the relationship between YAP and VGLL4 expression in tissular samples of HCC patients and found that YAP expression was negatively correlated with VGLL4 expression (Fig. 3E). Next, we calculated the YAP/VGLL4 ratio of each patient and found that the average value of YAP/VGLL4 ratios among all HCC patients were much higher compared with non-tumor patients (Fig. 3F). To further determine the potential effect of YAP/VGLL4 ratio in HCC, correlation analysis was performed. Similar to YAP, YAP/VGLL4 ratio was also positively correlated with serum 5-HT, tissular 5-HT and intraplatelet 5-HT per platelet respectively (Fig. 3G–I). Together, these results suggested that YAP/VGLL4 ratio was an important factor correlated with HCC prognosis. Moreover, it might be a potential diagnostic and prognostic marker for HCC patients.

### Clinical significance of 5-HT level and YAP/VGLL4 balance in HCC patients

**Figure 4:**
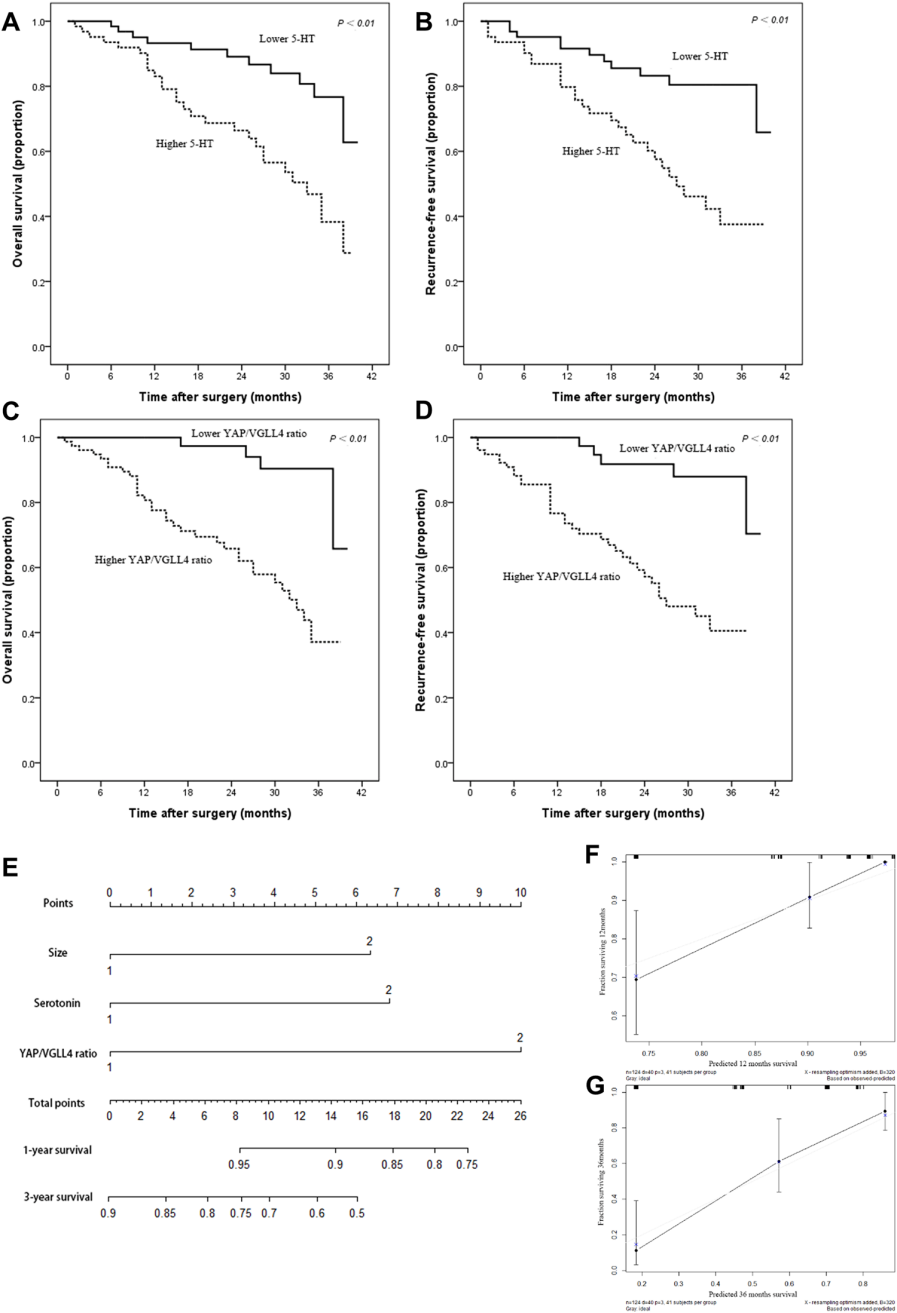
High 5-HT level and YAP/VGLL4 ratio were correlated with poor prognosis and indicated a low overall survival and short recurrence-free survival. (**A**,**B**) HCC patients with a higher level of 5-HT had poorer overall survival (**A**) and recurrence-free survival (**B**). (**C**,**D**) HCC patients with a higher YAP/VGLL4 ratio had poorer overall survival (**C**) and recurrence-free survival (**D**). (**E**–**G**) Nomogram for predicting the 1- and 3-year survival of HCC patients (**E**) and the calibration plot of the nomogram for 1 year (**F**) and 3 years (**G**). ns: no statistical significance, *P < 0.05, **P < 0.01.

The median of 5-HT level in serum and mean of YAP/VGLL4 ratio in tissue samples were determined as the cutoff value to evaluate the prognosis of HCC patients. Based on the analysis of clinical database, we found that high 5-HT level was prominently related to higher PLT (*P* < 0.001), larger tumor size (*P* < 0.001), more death (*P* = 0.002) and higher YAP/VGLL4 ratio (*P* = 0.016) and high YAP/VGLL4 ratio was related to higher ALP (*P* = 0.010), higher GGT (*P* < 0.001) and larger tumor size (*P* < 0.001), easier vascular invasion (*P* = 0.001), more death (*P* < 0.001) and a higher 5-HT level (*P* = 0.016) (Table 1). Additionally, ALP, GGT, tumor size, 5-HT level and YAP/VGLL4 ratio were all associated with overall survival and recurrence-free survival via univariate analysis (Table 2). Multivariate analysis demonstrated that only tumor size, 5-HT level and YAP/VGLL4 ratio were associated with overall survival and recurrence-free survival synchronously (Table 3). Survival analysis further revealed that higher level of 5-HT and YAP/VGLL4 ratio were both associated with a lower overall survival, shorter recurrence-free survival and poorer prognosis of HCC patients (Fig. 4A–D).

**Table 1:** Basic characteristics of HCC patients stratified according to the 5-HT level and YAP/VGLL4 balance.

Variable	Overall	5-HT			YAP/VGLL4 ratio		
		Lower (n = 62)	Higher (n = 62)	*P*	Lower (n = 47)	Higher (m = 77)	*P*
Sex, male/female	98/26	51/11	47/15	0.379	36/11	62/15	0.604
Age (ys)	51.1 ± 9.8	51.4 ± 9.5	50.8 ± 10.0	0.742	51.2 ± 11.3	51.1 ± 8.8	0.948
ALT (U/L)	47.5 (11–492)	44 (11–359)	52 (13–492)	0.615	41 (11–492)	53 (13–433)	0.460
AST (U/L)	46 (13–561)	41 (13–356)	53 (17–561)	0.119	40 (13–561)	48 (17–330)	0.070
ALP (U/L)	97 (26–388)	92 (26–241)	105 (56–388)	0.178	88 (36–173)	110 (26–388)	**0.010**
GGT (U/L)	69.5 (13–803)	59 (13–381)	83 (14–803)	0.235	44 (15–274)	90 (13–803)	**<0.001**
PLT (10^9^/L)	124 (25–417)	100 (25–227)	145 (32–417)	**<0.001**	112 (51–417)	132 (25–351)	0.476
INR (U/L)	1.1 (0.8–1.6)	1.1 (0.8–1.6)	1.1 (0.8–1.6)	0.161	1.1 (0.8–1.4)	1.1 (0.8–1.6)	0.327
ALB	39 (19–52)	38 (19–52)	39 (28–51)	0.503	39 (19–52)	39 (24–51)	0.513
AFP: ≥200/<200	62/55	26/32	36/23	0.079	19/25	43/30	0.099
Ascites: yes/no	14/110	5/57	9/53	0.256	3/44	11/66	0.177
Cirrhosis: yes/no	51/73	29/33	22/40	0.201	20/27	31/46	0.801
Tumor size: ≥5/<5 cm	56/68	16/46	40/22	**<0.001**	6/41	50/27	**<0.001**
Tumor number: multiple/single	30/94	14/48	16/46	0.675	7/40	23/54	0.059
Child-Pugh: A/B + C	111/13	57/5	54/8	0.379	44/3	67/10	0.244
Vascular invasion: yes/no	16/108	4/58	12/50	0.032	0/47	16/61	**0.001**
Death: yes/no	40/84	12/52	28/34	**0.002**	6/41	34/43	**<0.001**
YAP/VGLL4 ratio: high/low	77/47	32/30	45/17	**0.016**			
5-HT: high/low	62/62				17/30	45/32	**0.016**

**Table 2:** Univariate and multivariate analysis of factors associated with overall survival of HCC patients.

	uHR	*P*	aHR	*P*
Sex, male vs. female	0.696 (0.354–1.371)	0.295		
Age (ys)	1.002 (0.969–1.037)	0.888		
ALT (U/L)	1.001 (0.998–1.008)	0.354		
AST (U/L)	1.001 (0.997–1.004)	0.621		
ALP (U/L)	1.007(1.002–1.012)	**0.003**	1.006 (0.998–1.014)	0.122
GGT (U/L)	1.003 (1.001–1.005)	**0.015**	0.999 (0.994–1.003)	0.539
PLT (10^9^/L)	1.002 (0.997–1.006)	0.463		
INR	1.808 (0.216–15.151)	0.585		
ALB	0.985 (0.935–1.040)	0.589		
AFP, ≥200 vs. <200 (ng/ml)	1.012 (0.535–1.914)	0.971		
Ascites, yes vs. no	1.350 (0.528–3.450)	0.531		
Cirrhosis, yes vs. no	1.276 (0.675–2.410)	0.454		
Tumor size, ≥5 vs. <5 cm	4.201 (2.165–8.154)	**<0.001**	2.292 (1.091–4.812)	**0.028**
Tumor number, multiple vs. single	1.751 (0.878–3.490)	0.112		
Child-pugh, B + C vs. A	1.099 (0.390–3.096)	0.859		
Vascular invasion: yes vs. no	1.482 (0.622–3.535)	0.375		
5-HT, high vs. low	3.152 (1.594–6.234)	**0.001**	2.163 (1.056–4.433)	**0.035**
YAP/VGLL4 ratio, high vs. low	5.363 (12.225–12.926)	**<0.001**	2.944 (1.107–7.830)	**0.030**

**Table 3:** Univariate and multivariate analysis of factors associated with recurrence-free survival of HCC patients.

	uHR	*P*	aHR	*P*
Sex, male vs. female	0.680 (0.345–1.337)	0.264		
Age (ys)	1.006 (0.973–1.039)	0.735		
ALT (U/L)	1.000 (0.996–1.003)	0.800		
AST (U/L)	1.001 (0.997–1.004)	0.654		
ALP (U/L)	1.007 (1.003–1.012)	**0.002**	1.006 (0.999–1.014)	0.090
GGT (U/L)	1.003 (1.001–1.005)	**0.015**	0.999 (0.995–1.003)	0.639
PLT (10^9^/L)	1.001 (0.997–1.005)	0.714		
INR	1.490 (0.178–12.50)	0.713		
ALB	0.991 (0.939–1.046)	0.748		
AFP, ≥200 vs. <200 (ng/ml)	1.110 (0.589–2.092)	0.746		
Ascites, yes vs. no	1.423 (0.557–3.638)	0.461		
Cirrhosis, yes vs. no	1.192 (0.631–2.252)	0.588		
Tumor size, ≥5 vs. <5 cm	3.784 (1.953–7.333)	**<0.001**	2.229 (1. 066–4.661)	**0.033**
Tumor number, multiple vs. single	1.521 (0.770–3.006)	0.227		
Child-pugh, B + C vs. A	1.015 (0.361–2.850)	0.978		
Vascular invasion: yes vs. no	1.634 (0.682–3.910)	0.270		
5-HT, high vs. low	3.320 (1.651–6.674)	**0.001**	2.495 (1.194–5.215)	**0.015**
YAP/VGLL4 ratio, high vs. low	5.529 (2.269–13.473)	**<0.001**	3.269 (1.219–8.766)	**0.019**

### Predictive nomogram for survival

To predict the survival of HCC patients, prognostic nomogram was established via R software using independent risk factor consisting of tumor size, 5-HT level, YAP/VGLL4 ratio. Based on the nomogram, we can predict the one-year and three-year survival rate for HCC patients (Fig. 4E). In addition, calibration plots of the established nomogram were conducted and performed well with the ideal model (Fig. 4F,G). On the basis of these results, it indicted that higher total point of the nomogram mean poorer prognosis of HCC patients.

## Discussion

Nowadays, serotonin in the central nervous system is well studied. Besides, peripheral serotonin also plays an essential role in regulating organ system, especially in promoting tumor process and cell proliferation. Our previous study have demonstrated that serotonin promotes malignant biological behavior of hepatocellular carcinoma via regulating YAP expression^7^. However, how the HCC process affected by YAP expression is still confusing. In this study, we demonstrated that 5-HT level affected the HCC prognosis via regulating YAP/VGLL4 balance. 5-HT and YAP/VGLL4 balance were closely related with prognosis of HCC patients. Moreover, established nomogram has the ability to accurately predict the survival of HCC patients.

Accumulated studies indicated that 5-HT acted as a mitogen in different kinds of cancer^7^. Starlinger *et al*. study demonstrated that 5-HT was a relevant inducer in liver regeneration^13^. Additionally, studies conducted by Soll *et al*. and Liang *et al*. also revealed that 5-HT promoted hepatoma cell proliferation. Similarly, our previous study demonstrated that serum 5-HT level, platelets, intraplatelet 5-HT and platelet extract 5-HT in HCC patients were all higher. On the contrary, there was no statistical significance in plasma 5-HT between the two groups. Because decoagulant was used during the plasma preparation process, platelets were inactivated and 5-HT were still retained in platelets. Oppositely, the platelets were activated during the process of serum preparation, and 5-HT were released from the activated platelets. As a result, 5-HT level was higher only in serum between the two groups. Similarly, the calculated intraplatelet 5-HT per platelet and platelet extract 5-HT per platelet were both higher and positively correlated in HCC patients. Moreover, higher 5-HT level was significantly associated with a decrease overall survival and disease-free survival.

As an oncogene, YAP can promote proliferation and migration of diverse cancer cells via activating target genes, such as CTGF, AREG and *et al*.^14–17^. Though YAP does not contain any DNA-binding domains, it interacts with TEADs to activate target genes of YAP. Notably, recent researches demonstrated that VGLL4, a member of the Vestigial-like proteins, has emerged as a new TEAD-interacting factor^8,9^. An increasing number of studies revealed VGLL4 inhibited proliferation by disrupting YAP-TEAD interaction and targeting TEAD for degradation^8–10,18,19^. In addition, a study conducted by Li *et al*. indicated that VGLL4 suppressed EMT via Wnt/β-catenin pathway in gastric cancer^10^. Accumulating studies have reported that YAP and VGLL4 function as regulators in tumor playing opposite effects via competitive inhibition. Thereupon, YAP/VGLL4 balance might play an important role in tumorigenesis and prognosis. A study conducted by Jiao *et al*. demonstrated that YAP/VGLL4 ratio were sharply skewed and well correlated with tumor progression in gastric cancer^8^. Similar results were obtained by Zhang *et al*. and Lin *et al*. in lung cancer and postnatal cardiac growth respectively^9,18^. Our previous study also demonstrated YAP expression was regulated by 5-HT in hepatoma cells^7^. However, whether YAP/VGLL4 balance was correlated with hepatocellular carcinoma and regulated by 5-HT was poorly understood. In our study, we found that YAP expression was up-regulated in HCC patients. Oppositely, VGLL4 expression was down-regulated. We further showed that 5-HT level was positively correlated with YAP expression and negatively correlated with VGLL4 expression severally. In addition, YAP/VGLL4 ratio was higher in HCC patients and positively correlated with 5-HT level. Further study conducted by us demonstrated that high 5-HT level and high YAP/VGLL4 ratio were both significantly associated with a decrease overall survival, disease-free survival and a poor prognosis. Moreover, the mechanism study which is ongoing indicated that serotonin promoted YAP expression and hepatoma cell proliferation. Meanwhile, the VGLL4 expression was suppressed by serotonin administration. Additionally, YAP-siRNA suppressed the proliferation promotion effect induced by serotonin. Though YAP expression was down-regulated by YAP-siRNA, the VGLL4 expression was not affected by it (supplementary materials). Taken together, 5-HT level might affect prognosis of hepatocellular carcinoma via regulating the YAP/VGLL4 balance. The results was also supported by a study on breast cancer^20^.

Through the analysis of clinical database, 5-HT level was found to be associated with PLT, tumor size, death and YAP/VGLL4 ratio and YAP/VGLL4 ratio was correlated with ALP, GGT, tumor size, vascular invasion, death and the 5-HT level. However, AFP, cirrhosis and Child-Pugh system were not correlated with 5-HT and YAP/VGLL4 ratio. Because serotonin and YAP/VGLL4 ratio were used to predict the prognosis of HCC in our study. However, AFP was used to diagnosis HCC not to predict the prognosis. Thus, there was no statistical significance between high 5-HT group and low 5-HT group in AFP level. Additionally, there was no study demonstrated that cirrhosis and Child-Pugh was associated with the prognosis of HCC. Additionally, only 124 patients were selected in our study and the characteristics of the selected patients might also affect the factors which were associated with prognosis. Based on the univariate and multivariate analysis of factors associated with overall and recurrence-free survival, nomogram was established. Using the nomogram, we can accurately predict the survival of HCC patients. Besides used in HCC, nomogram has been used to predict the survival of papillary thyroid carcinoma, periampullary adenocarcinoma, esophageal Adenocarcinoma, gastric cancer, non-small cell lung cancer and hepatocellular carcinoma^21–26^.

Our study firstly explored regulating effect of 5-HT on YAP/VGLL4 balance. We further explored the relationship of 5-HT, YAP/VGLL4 balance and prognosis. Additionally, the nomogram based on tumor size, 5-HT and YAP/VGLL4 balance were established in our study. Using the nomogram, we could accurately predict prognosis of HCC patients. Moreover, regulating YAP/VGLL4 balance might be a potential therapeutic method for HCC patients in the further.

## Materials and Methods

### Patients

From January 2014 to June 2015, a total of 124 HCC patients and 21 hepatic hemangioma patients undergoing partial hepatectomy at the The Second Xiangya Hospital of Central South University were selected in our study. The inclusion criteria of tumor group were as follows: (1) First diagnosed and meet the National Comprehensive Cancer Network (NCCN) criteria of hepatocellular carcinoma, (2) Not received radiotherapy, chemotherapy before hepatectomy, (3) there were no co-occurrences of other cancers, (4) not received splenectomy, (5) not use medication and not suffered from diseases that affected the function and count of platelet. The inclusion criteria for non-tumor group was same with tumor group expected for the first criteria.

For each patient, the clinical and pathologic data was gathered via electronic medical records as follow: age, gender, aspartate transaminase (AST), alanine aminotransferase (ALT), alkaline phosphatase (ALP), γ-glutamyl transpeptidase (γ-GGT), albumin (ALB), international normalized ratio (INR), platelet count (PLT), alpha-fetoprotein (AFP), ascites, cirrhosis, tumor sizes, tumor number, Child-Push, vascular invasion, pathologic reports and death.

Follow-up survey was conducted by two independent investigators, who had no knowledge of the study. All of the patients were follow up every 3 months until April 2017. Patient who lost touch with us for four times in succession would be excluded in our study. All selected patients were treated based on the Clinical Practice Guidelines for Hepatocellular Carcinoma established by the Japanese Society of Hepato-Biliary-Pancreatic Surgery. Before analysis of blood samples and patient data in our study, the informed consent from the patients and the approval from the Central South University Ethics Committee were both obtained. Study was performed in ac cordance with the ethical standards of the Committee on Human Experimentation and the Helsinki Declaration.

### Sample preparation

Routine blood samples as well as serum and plasma preparation were conducted before the surgery. Additionally, tissue samples of all patients were collected during the operation.

Notably, 5-HT is stored in alpha granules of platelet. During the preparation of blood samples, the 5-HT might be released by artificial activation of platelets *in vitro*. Thus, an optimized technique was applied base on Starlinger’s study^2^. Briefly, blood sample was drawn into CTAD tube and placed on ice immediately. Then the sample was subject to an initial centrifugation at 1000 ∗ g in 4 °C for 10 minutes and following centrifugation was conducted at 10000 ∗ g in 4 °C for 10 minutes to remove remaining platelets. Finally, the supernatant was stored at −80 °C^2^.

Serum sample of selected patients was collected without addition of anti-coagulants and centrifuged at 1000 ∗ g in room temperature for 30 minutes. The supernatant was collected and also stored at −80 °C.

In order to obtain the platelet extract, the blood sample was drawn into tube with trisodium citrate and centrifuged at 125 ∗ g for 20 minutes. Then the supernatant was wash by HEPES-tyrode buffer for three times. Finally, the washed platelets were resuspended in lysis buffer to generate platelet extract^2^.

### Quantification of 5-HT

The level of 5-HT in serum, plasma and platelet extracts were measured via human 5-HT ELISA Kit according to the manufacturer’s instruction. Because the serum 5-HT level was the whole 5-HT which was released after platelet activation, and the plasma 5-HT level was the circulating amount of 5-HT. The actual level of intraplatelet 5-HT level was calculated by subtracting the plasma 5-HT level from the serum 5-HT level. The intraplatelet 5-HT per platelet was calculated as dividing intraplatelet 5-HT level by platelet counts. Similarly, the platelet extract 5-HT per platelet was calculated as dividing the platelet extract 5-HT level by platelet counts.

### RNA isolation and quantitative real-time PCR (qRT-PCR)

TRIzol reagent (Invitrogen, Carlsbad, CA, USA) was to extract the total RNA from tissue samples. The SYBR Premix Ex Taq Kit (Takara, Tokyo, Japan) and TaqMan microRNA assays (Applied Biosystems, Foster City, CA, USA) were used to conduct qRT-PCR. The RNA expression was calculated via the relative quantification method. The primers were shown as follow.

YAPF: 5′-GCTACAGTGTCCCTCGAACC-3′

R: 5′-CCGGTGCATGTGTCTCCTTA-3′

VGLL4F: 5′-AACTGCAACCTCTCGCACTG-3′

R: 5′-GAGTGGGTGTCGCTGTTGAA-3′

GAPDHF: 5′-CGCGAGAAGATGACCCAGAT-3′

R: 5′-GCACTGTGTTGGCGTACAGG-3′

### Western blot analysis

Proteins were extracted from the tissue samples according to the manufacturer’s instruction. Western blot analysis were conducted as previously described^27^. The antibodies used in the study were shown as follow: anti-YAP (ab205270, Abcam, Cambridge, MA, USA), VGLL4 (ab140290, Abcam, Cambridge, MA, USA).

### Statistical analysis

The data were expressed as mean ± standard deviation (SD). The correlation differences between the respective groups were evaluated by either analysis of variance (ANOVA) or nonparametric test, as applicable, using SPSS v20 (SPSS, IBM Inc.). The correlation between 5-HT level and YAP expression, 5-HT level and VGLL4 expression, YAP expression and VGLL4 expression, 5-HT level and YAP/VGLL4 ratio were evaluated by Pearson’s correlation analysis. Because the amount of selected patient was 124, the optimal cut-off level of 5-HT was determined as median rather than receiver operating curve (ROC) analysis. The cut-off of YAP/VGLL4 ratio was determined as mean, because the value of YAP/VGLL4 ratio could be considered to obey normal distribution. Overall survival was calculated as the time from the date of hepatectomy until death or the last follow-up survey. Recurrence-free survival was calculated as the time from the date of hepatectomy until death, tumor recurrence or end of the follow-up. In addition, the survival curves were plotted by K-M method. A nomogram for the 5-HT level and YAP/VGLL4 ratio, which were associated with survival, was established using rms package of R software (R 3.2.1 software, Institute for Statistics and Mathematics). The selected factors in nomogram were based on multivariate analysis of factors which was associated with overall survival and recurrence-free survival of HCC patients. Calibration plots were conducted to evaluate the performance characteristics of the predictive nomogram. *P*-value less than 0.05 was considered statistically significant.

## Electronic supplementary material

Supplementary figure

## References

[1] Ferenci P (2010). Hepatocellular carcinoma (HCC): a global perspective. J Clin Gastroenterol.

[2] Lesurtel M, Clavien PA (2014). Platelet-derived serotonin: translational implications for liver regeneration. Hepatology.

[3] Soll C (2010). Serotonin promotes tumor growth in human hepatocellular cancer. Hepatology.

[4] Liang C (2013). Serotonin promotes the proliferation of serum-deprived hepatocellular carcinoma cells via upregulation of FOXO3a. Molecular cancer.

[5] Zhang L (2015). The hippo pathway effector YAP regulates motility, invasion, and castration-resistant growth of prostate cancer cells. Molecular and cellular biology.

[6] Kowalik MA (2011). Yes-associated protein regulation of adaptive liver enlargement and hepatocellular carcinoma development in mice. Hepatology.

[7] Liu, S. *et al*. Effects and related mechanisms of serotonin on malignant biological behavior of hepatocellular carcinoma via regulation of Yap. *Oncotarget*, 10.18632/oncotarget.17658 (2017). 10.18632/oncotarget.17658 PMC5564575 28537892

[8] Jiao S (2014). A peptide mimicking VGLL4 function acts as a YAP antagonist therapy against gastric cancer. Cancer cell.

[9] Zhang W (2014). VGLL4 functions as a new tumor suppressor in lung cancer by negatively regulating the YAP-TEAD transcriptional complex. Cell Res.

[10] Li H (2015). VGLL4 inhibits EMT in part through suppressing Wnt/beta-catenin signaling pathway in gastric cancer. Medical oncology.

[11] Jiao S (2017). VGLL4 targets a TCF4-TEAD4 complex to coregulate Wnt and Hippo signalling in colorectal cancer. Nature communications.

[12] Jiang W (2015). Downregulation of VGLL4 in the progression of esophageal squamous cell carcinoma. Tumour biology: the journal of the International Society for Oncodevelopmental Biology and Medicine.

[13] Starlinger P (2014). Evidence for serotonin as a relevant inducer of liver regeneration after liver resection in humans. Hepatology.

[14] Zhao B (2008). TEAD mediates YAP-dependent gene induction and growth control. Genes & development.

[15] Hao Y, Chun A, Cheung K, Rashidi B, Yang X (2008). Tumor suppressor LATS1 is a negative regulator of oncogene YAP. The Journal of biological chemistry.

[16] Perra A (2014). YAP activation is an early event and a potential therapeutic target in liver cancer development. Journal of hepatology.

[17] Wang J (2013). Mutual interaction between YAP and CREB promotes tumorigenesis in liver cancer. Hepatology.

[18] Lin, Z. *et al*. Acetylation of VGLL4 Regulates Hippo-YAP Signaling and Postnatal Cardiac Growth. *Developmental cell*, 10.1016/j.devcel.2016.09.005 (2016). 10.1016/j.devcel.2016.09.005 PMC5121000 27720608

[19] Tajonar A (2013). Brief report: VGLL4 is a novel regulator of survival in human embryonic stem cells. Stem Cells.

[20] Zhang Y (2017). VGLL4 Selectively Represses YAP-Dependent Gene Induction and Tumorigenic Phenotypes in Breast Cancer. Sci Rep.

[21] Jianyong L, Zhihui L, Rixiang G, Jingqiang Z (2018). Using a nomogram based on preoperative serum fibrinogen levels to predict recurrence of papillary thyroid carcinoma. BMC cancer.

[22] He C (2018). Nomograms predict long-term survival for patients with periampullary adenocarcinoma after pancreatoduodenectomy. BMC cancer.

[23] Goense, L. *et al*. Preoperative Nomogram to Risk Stratify Patients for the Benefit of Trimodality Therapy in Esophageal Adenocarcinoma. *Annals of surgical oncology*, 10.1245/s10434-018-6435-4 (2018). 10.1245/s10434-018-6435-4 PMC5928173 29569125

[24] Narita Y (2018). Establishment and validation of prognostic nomograms in first-line metastatic gastric cancer patients. J Gastrointest Oncol.

[25] Mao Q (2018). A nomogram to predict the survival of stage IIIA-N2 non-small cell lung cancer after surgery. J Thorac Cardiovasc Surg.

[26] Hsu CY (2018). Using nomogram of the Barcelona Clinic Liver Cancer system for treatment selection in patients with stage C hepatocellular carcinoma. BMC cancer.

[27] Liu S (2016). The Protective Role of Curcumin in Zymosan-Induced Multiple Organ Dysfunction Syndrome in Mice. Shock.

