# Supplementary material for: Serotonin and YAP/VGLL4 Balance Correlated with Progression and Poor Prognosis of Hepatocellular Carcinoma
Source: Sci Rep. 2018 Jun 27;8:9739. doi: 10.1038/s41598-018-28075-9 (PMC6021381; doi:10.1038/s41598-018-28075-9)
Supplement: Supplementary file 1 — Supplementary figure [file 41598_2018_28075_MOESM1_ESM.docx]

Serotonin and YAP/VGLL4 Balance Correlated with Progression and Poor Prognosis of Hepatocellular Carcinoma

Bo Shu^1^, Mimi Zhai^2^, Xiongying Miao^1^, Chao He^1^, Chaolin Deng^1^, Yu Fang^1^, Ming Luo^1^, Luyao Liu^1^, Sushun Liu^1^

^1^Department of General Surgery, the Second Xiangya Hospital, Central South University, Changsha, Hunan 410011, China

^2^Department of Hepatobiliary Surgery, the First Affiliated Hospital of Xi’an Jiaotong University, Xi’an, Shaanxi 710061, China

Author contributions: Shu B and Zhai Mimi analyzed the data and wrote the manuscript; Miao XY and Fang Y analyzed the data; He C, Deng CL and Liu LY created the figures; Ming Luo revised the manuscript; Liu SS designed study, contributed discussion and edited the manuscript as corresponding author.

Correspondence to: Sushun Liu, MD, Department of General Surgery, The Second Xiangya Hospital, Central South University, Changsha, Hunan 410011, China. No. 139 Renmin Road, Changsha 410011, Hunan Province, China. [surun9566@126.com](mailto:surun9566@126.com) or [surun9566@csu.edu.cn](mailto:surun9566@csu.edu.cn)

Telephone: +86-731-85295120 Fax: +86-731-85533525


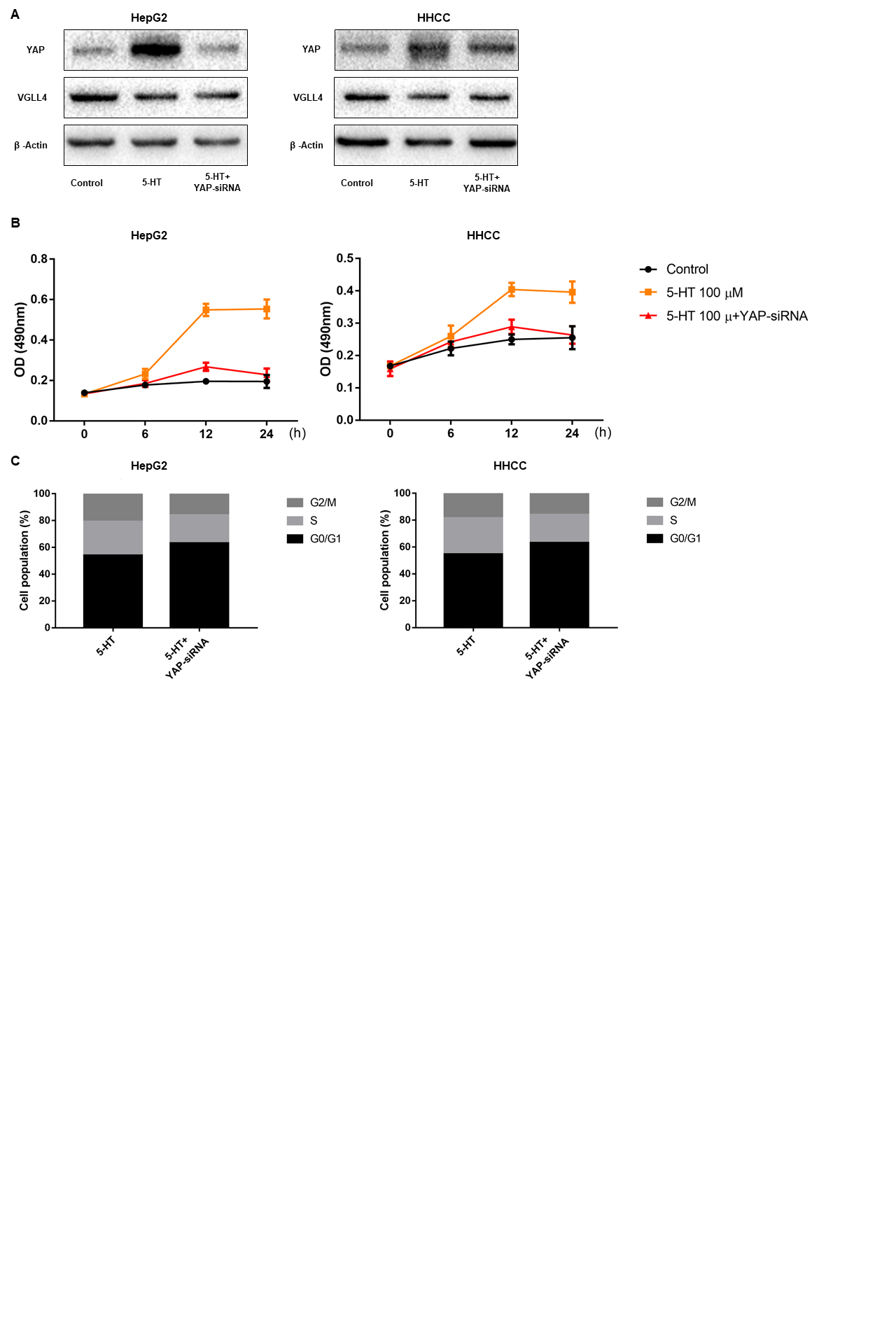


Supplementary figure. Serotonin promoted YAP expression and hepatoma cell proliferation. Meanwhile, the VGLL4 expression was suppressed by serotonin administration. Additionally, YAP-siRNA suppressed the proliferation promotion effect induced by serotonin. Though YAP expression was downregulated by YAP-siRNA, the VGLL4 expression was not affected. In conclusion, 5-HT might contribute to the progression and poor prognosis of HCC via regulating YAP/VGLL4 balance.
